# Biofilm as a production platform for heterologous production of rhamnolipids by the non-pathogenic strain *Pseudomonas putida* KT2440

**DOI:** 10.1186/s12934-016-0581-9

**Published:** 2016-10-24

**Authors:** Vinoth Wigneswaran, Kristian Fog Nielsen, Claus Sternberg, Peter Ruhdal Jensen, Anders Folkesson, Lars Jelsbak

**Affiliations:** 1 Department of Systems Biology, Technical University of Denmark, 2800 Kgs. Lyngby, Denmark; 2 National Food Institute, Technical University of Denmark, 2800 Kgs. Lyngby, Denmark; 3 National Veterinary Institute, Technical University of Denmark, 1870 Frederiksberg C, Denmark

**Keywords:** Rhamnolipids, Biosurfactants, Heterologous biosynthesis, *Pseudomonas putida* KT2440, Biofilm, Synthetic promoter library, UHPLC-HRMS, UHPLC-MS/HRMS

## Abstract

**Background:**

Although a transition toward sustainable production of chemicals is needed, the physiochemical properties of certain biochemicals such as biosurfactants make them challenging to produce in conventional bioreactor systems. Alternative production platforms such as surface-attached biofilm populations could potentially overcome these challenges. Rhamnolipids are a group of biosurfactants highly relevant for industrial applications. However, they are mainly produced by the opportunistic pathogen *Pseudomonas aeruginosa* using hydrophobic substrates such as plant oils. As the biosynthesis is tightly regulated in *P. aeruginosa* a heterologous production of rhamnolipids in a safe organism can relive the production from many of these limitations and alternative substrates could be used.

**Results:**

In the present study, heterologous production of biosurfactants was investigated using rhamnolipids as the model compound in biofilm encased *Pseudomonas putida* KT2440. The *rhlAB* operon from *P. aeruginosa* was introduced into *P. putida* to produce mono-rhamnolipids. A synthetic promoter library was used in order to bypass the normal regulation of rhamnolipid synthesis and to provide varying expression levels of the *rhlAB* operon resulting in different levels of rhamnolipid production. Biosynthesis of rhamnolipids in *P. putida* decreased bacterial growth rate but stimulated biofilm formation by enhancing cell motility. Continuous rhamnolipid production in a biofilm was achieved using flow cell technology. Quantitative and structural investigations of the produced rhamnolipids were made by ultra performance liquid chromatography combined with high resolution mass spectrometry (HRMS) and tandem HRMS. The predominant rhamnolipid congener produced by the heterologous *P. putida* biofilm was mono-rhamnolipid with two C_10_ fatty acids.

**Conclusion:**

This study shows a successful application of synthetic promoter library in *P. putida* KT2440 and a heterologous biosynthesis of rhamnolipids in biofilm encased cells without hampering biofilm capabilities. These findings expands the possibilities of cultivation setups and paves the way for employing biofilm flow systems as production platforms for biochemicals, which as a consequence of physiochemical properties are troublesome to produce in conventional fermenter setups, or for production of compounds which are inhibitory or toxic to the production organisms.

**Electronic supplementary material:**

The online version of this article (doi:10.1186/s12934-016-0581-9) contains supplementary material, which is available to authorized users.

## Background

Rhamnolipids is a group of biosurfactants with noticeable industrial potential. Their low toxicity and biodegradability combined with their potent surface tension reducing and emulsifying activity has made them one of the most studied biosurfactants. Their possible applications range from industry, agriculture and bioremediation to personal care and medicine [1, 2]. Rhamnolipids were first described by Jarvis and Johnson [3] and are produced by various organisms but mainly known from the opportunistic pathogen *Pseudomonas aeruginosa* in which most studies have been made.

The rhamnolipids encompass a diverse group of compounds composed of one or two rhamnose molecules linked to one or two β-hydroxy fatty acids by β-glycosidic bonds. The rhamnose and fatty acid composition depends on strain and growth conditions [4, 5]. The most abundant rhamnolipid congener produced by *P. aeruginosa* is the di-rhamnolipid l-rhamnosyl-l-rhamnosyl-3-hydroxydecanoyl-3-hydroxydecanoate (Rha-Rha-C_10_-C_10_) [4].

The rhamnose moiety in rhamnolipids is synthesised from glucose [6, 7] and the fatty acid is provided from de novo synthesis [8]. Three enzymes mediate the synthesis of rhamnolipids in *P. aeruginosa*. The first step is the synthesis of 3-(3-hydroxyalkanoyloxy)alkanoate (HAA) mediated by RhlA [8, 9]. HAA is the lipidic precursor that together with dTDP-l-rhamnose is the substrate of RhlB for synthesising mono-rhamnolipids [10]. The rhamnosyltransferase II (*rhlC*) is responsible for addition of another rhamnose moiety to make di-rhamnolipid [10]. The *rhlA* and *rhlB* genes constitute an operon [11, 12] while *rhlC* is placed in another part of the genome [10]. Expression of the *rhlAB* operon in *P. aeruginosa* is highly regulated at multiple levels and subjected to both quorum sensing control as well as regulation by environmental factors such as phosphate, nitrate, ammonium and iron availability [9, 11, 13–16].

The complex regulation of rhamnolipid synthesis makes it difficult to control bioproduction. Furthermore, *P. aeruginosa* is an opportunistic pathogen. To overcome these constraints various studies have been made in order to produce rhamnolipids in a heterologous host such as *Escherichia coli*, *Pseudomonas fluorescens*, *Pseudomonas oleovorans* and *Pseudomonas putida* [8, 17, 18] by introducing the *rhlAB* operon. The ability of the host to tolerate rhamnolipid production can be a challenge as these molecules hamper growth at high concentrations [18]. However, by using a species from the same genus a high inherent tolerance can be achieved as shown for *P. putida* [17, 18].

The heterologous rhamnolipid production in *P. putida* KT2440 has so far been examined in conventional bioreactor systems with planktonic cells [17, 18]. This type of growth is associated with difficulties owing to foam formation caused by aeration of the culture [19–21]. We hypothesize that cultivation of cells in a surface attached biofilm can reduce this problem. In contrast to planktonic growth, biofilms represent a sessile mode of growth, and cells growing in a biofilm have a lowered growth activity compared to their planktonic counterparts [22]. In connection to rhamnolipid production this phenotype is desirable as production is growth independent and growth should be minimised in order to achieve high rhamnolipid yields [18, 19].

In the present study we use *P. putida* KT2440 biofilm as a production platform for heterologous production of rhamnolipids by constructing a synthetic promoter library (SPL). We show that rhamnolipid production in a biofilm eliminates the formation of foam, which in other production setups result in significant challenges. A synthetic promoter library was designed and constructed to obtain various expression levels of rhamnolipid synthesis and to evaluate the effect of biosurfactant production on cells and on biofilm capabilities. A method for characterisation and quantification of the produced rhamnolipids was developed based on ultra-high performance liquid chromatography (UHPLC) combined with high resolution (HRMS) and tandem mass spectrometry (MS/HRMS).

## Results

### Using a synthetic promoter library to modify expression of *rhlAB* in *P. putida*

The native *rhlAB* promoter is controlled by the RhlI/RhlR quorum sensing system in *P. aeruginosa,* and it has been reported that the *rhlI* and *rhlR* genes are required for activity of the native *rhlAB* promoter in heterologous hosts [13]. To bypass the need for quorum sensing to achieve high expression levels, and to evaluate the influence of rhamnolipid production on cells and biofilm formation, we engineered a series of strains capable of producing rhamnolipids at different levels in a quorum sensing independent manner using a SPL [23] to drive expression of the *rhlAB* biosynthesis genes.

**Fig. 1:**
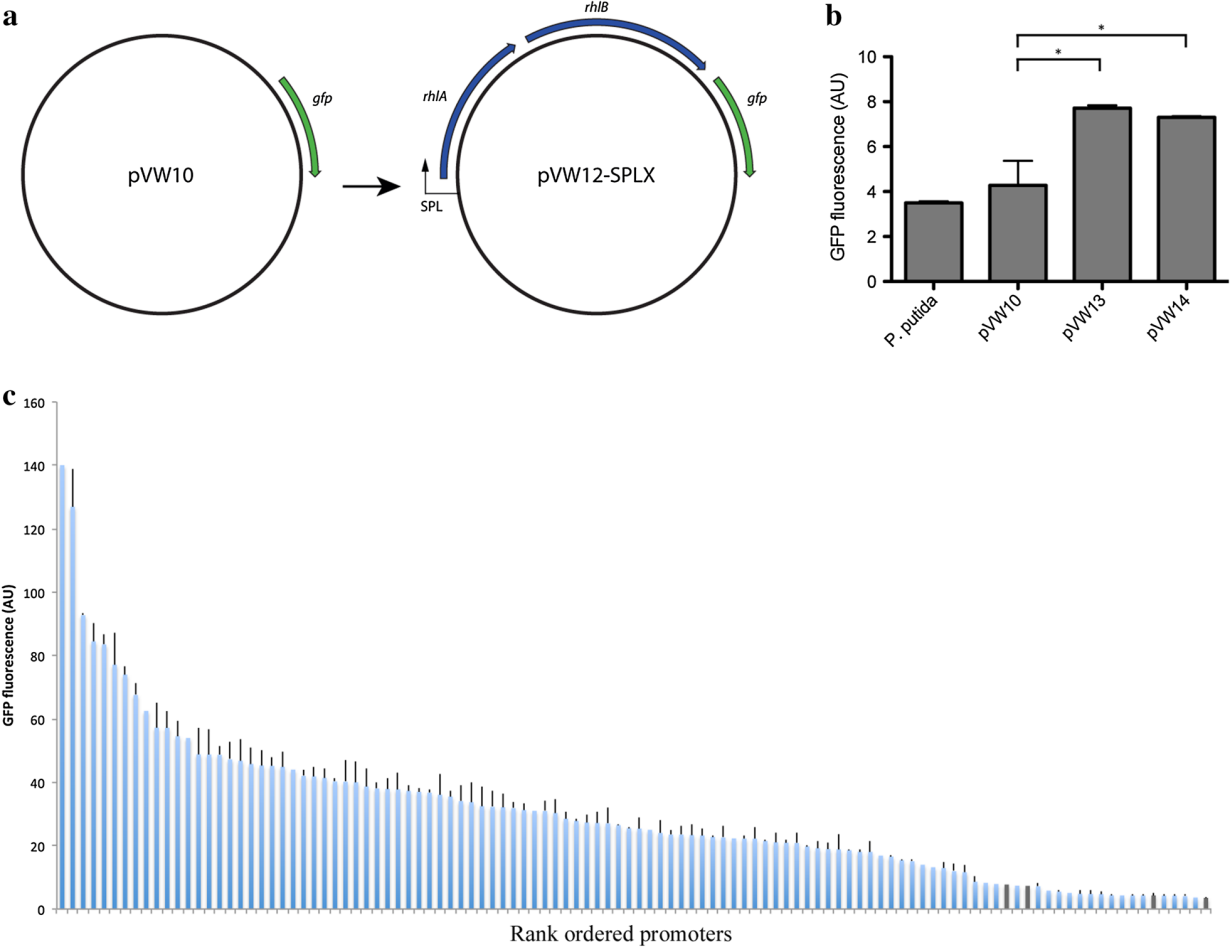
Construction and utilization of a synthetic promoter library in *P. putida*. **a** Outline of the plasmid construction strategy. The genes *gfp*, *rhlA* and *rhlB* indicate the green fluorescence protein, rhamnosyltransferase chain A and rhamnosyltransferase chain B, respectively. SPL indicate synthetic promoter library. The X in pVW12-SPLX represents any of the 120 synthetic promoters constructed in this study (see “Methods” section). **b** Gfp fluorescence measurement in a series of control strains without SPL. The control strains are wild type strain (*P. putida*) and strains containing an empty vector (pVW10), plasmid containing *rhlAB* operon (pVW13) with no promoter and the *rhlAB* operon with the native promoter from PAO1 (pVW14). The *asterisks* represent a significant difference (*P* < 0.05). **c** The different promoter strengths in the constructed SPL determined as Gfp intensities. The controls strains depicted in **b** are shown in *grey*. The *columns* represent mean value and the *error bars* indicate standard deviation. The Gfp intensities have been replicated 1–3 times. An enlarged picture of **c** can be found in Additional file 1: Figure S1 in which the name of the constructed promoters is shown

The synthetic promoter library was constructed on the basis of 16S rRNA promoter sequences from *P. putida* KT2440 and *P. aeruginosa* PAO1 in order to achieve strong constitutive promoters [24]. The SPL was designed by preserving the consensus sequences and degenerating the surrounding bases to modulate promoter strength as previously described [23, 25]. The SPL was placed in front of the *rhlAB* operon to achieve varying expression levels of the rhamnolipid biosynthesis genes (Fig. 1a). A *gfp* reporter was placed downstream of the *rhlAB* operon to enable determination of promoter activities and indirect monitoring of the rhamnolipid biosynthesis.

The wild type strain and a strain harbouring the *gfp* reporter vector without a promoter (pVW10) showed no significant difference in fluorescence intensity (Fig. 1b). The promoter activity of the *rhlAB* operon without SPL (pVW13) or with the native promoter from PAO1 (pVW14) showed a small but significant increase in activity compared to the reference vector, indicating an inherent but low promoter activity ascribed to the *rhlAB* operon (Fig. 1b). However, in these two cases rhamnolipid production could not be detected (data not shown). The constructed SPL resulted in a diverse set of synthetic promoters with varying strength (Fig. 1c). The majority of the synthetic promoters supported higher *gfp* expression levels than the control strains. An investigation of the SPL activity revealed a distribution of expression levels with most promoters between 10 and 50 AU.

### Gfp expression correlates with rhamnolipid production

**Fig. 2:**
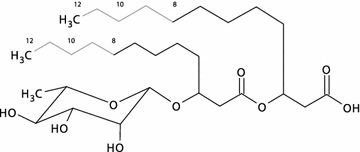
Outline of the different rhamnolipid congeners. The *grey part* of the structure is the varying part of the molecule. The *numbers* indicate the length of the fatty acid side chain. The most predominant congener produced by the recombinant *P. putida* is Rha-C_10_-C_10_. The different rhamnolipid congeners were determined based on their elemental composition

The synthetic promoter strength determined by Gfp fluorescence should reflect a corresponding expression of the *rhlAB* operon (Fig. 1a). To evaluate the correlation between fluorescence intensity and *rhlAB* expression we analysed rhamnolipid production in a selection of strains with synthetic promoters of different strengths. To this end, we developed a method based on ultra performance liquid chromatography (UHPLC) combined with high resolution mass spectrometry (HRMS) and tandem HRMS for simultaneous quantitative and qualitative analysis of the produced rhamnolipids (Additional file 1: Figure S4, Table S2). The rhamnolipid congeners were identified and quantified based on their elemental composition (Fig. 2) and were verified by positive and negative electrospray ionisation in UHPLC-HRMS. Although a mixture of rhamnolipids could be produced by *P. putida*, the predominant congener was Rha-C_10_-C_10_ followed by Rha-C_10_-C_12_, Rha-C_10_-C_12:1_ and Rha-C_8_-C_10_ (Additional file 1: Figure S4). The structural composition was elucidated by MS/HRMS fragmentation pattern (Additional file 1: Table S2). These results are in accordance with previous results [4, 26]. The elution pattern supported the determined structural composition.

**Fig. 3:**
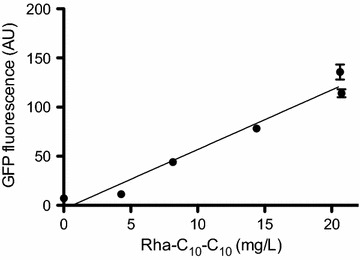
Correlation between Rha-C_10_-C_10_ concentration and *gfp* expression. The linear correlation between *gfp* and Rha-C_10_-C_10_ enable fluorescence to be used as an indirect measure of rhamnolipid biosynthesis

Analysis of rhamnolipid production in a selection of six strains with different synthetic promoter strengths revealed a clear linear correlation between Gfp activity and Rha-C_10_-C_10_ concentration (Fig. 3). Hence, Gfp fluorescence can be used as an indirect measure of rhamnolipid production.

### The cost of producing rhamnolipids

**Fig. 4:**
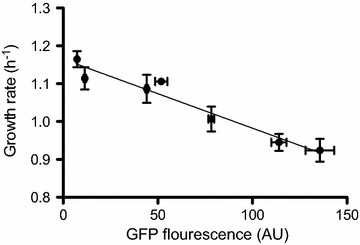
Correlation between rhamnolipid production and growth rate. Biosynthesis of rhamnolipids has an impact on planktonic growth rate. The cells grow slower as they produce more rhamnolipids

The wide range of synthetic promoter strengths made it possible to investigate the metabolic load associated with rhamnolipid production. An initial screening of the constructed strains showed an evident correlation between expression level and the growth rate (Additional file 1: Figure S2). The observation that increased rhamnolipid production was correlated to a reduction in growth rate was confirmed by analysis of a sub-set of promoters that covered the SPL (Fig. 4). Specifically, we found that a tenfold increase in rhamnolipid production results in a 14% lower growth rate.

### Heterologous expression of rhamnolipids in *P. putida* results in enhanced biofilm production

**Fig. 5:**
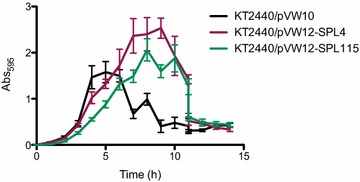
Biofilm development in microtiter plate assays. The amount of biofilm was quantified for the reference strain (*black*), a medium rhamnolipid producer (*red*) and a high rhamnolipid producer (*green*) at the specified time points. Each data point represents the mean and the *error bars* indicate standard deviations of eight replicates

Rhamnolipids are biosurfactants that may potentially interfere with *P. putida* biofilm formation [27]. To investigate the effect of rhamnolipid production on biofilm capabilities of the engineered strains, we used microtiter plate based assays to measure biofilm formation as a function of time for strains with high (KT2440/pVW12-SPL115) or medium (KT2440/pVW12-SPL4) rhamnolipid production which had Gfp fluorescence levels at 136 and 52 AU respectively. In these assays, the biofilm development of all strains followed a similar biofilm developmental progress with dispersal of the biofilm upon reaching their maximal quantity (Fig. 5) as previously described for *P. putida* biofilm formation [28]. Surprisingly, the rhamnolipid producing strains had an enhanced biofilm production and reached a higher biomass compared to the reference strain. The decrease in planktonic growth rate (Fig. 4) was reflected in a slower biofilm development of the high rhamnolipid producer although a higher biomass than the reference strain was eventually reached (Fig. 5). The medium rhamnolipid producer and the reference strain follow the same biofilm development until the dispersal of the reference strain. Hereafter, the rhamnolipid producing strain continues to grow and reach 60% more biomass than the reference strain. Although both of the rhamnolipid producing strains reached a higher biofilm biomass, the biofilm dispersal initiates earlier in the high rhamnolipid producer than the medium rhamnolipid producer. Hence, the amount of present rhamnolipids does have an impact on dispersal, however, compared to the reference strain rhamnolipid producers reach higher biofilm biomass.

### Heterologous expression of rhamnolipids in *P. putida* increase swarming motility

**Fig. 6:**
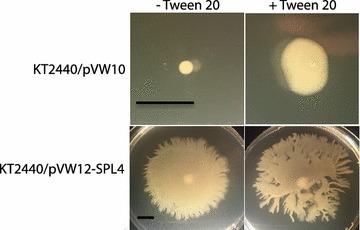
Effect of rhamnolipid and the surfactant Tween 20 on swarming motility. The *top row* is the reference strain (KT2440/pVW10) and the *bottom row* is a rhamnolipid producer (KT2440/pVW12-SPL4). The *right column* has been added 0.0005% Tween 20 and the *left column* have not. The *scale bars* indicate the size of 1 cm on the pictured strain. The presence of Tween 20 increased the swarming motility of both strains but in particular for the reference strain

Rhamnolipids have been shown to have an effect on both swarming motility and biofilm formation in *P. aeruginosa* [29], and similar motility-related effects could potentially explain the observation of altered biofilm formation in our engineered *P. putida* strains. Indeed, we observed that heterologous expression of rhamnolipids in *P. putida* results in an increase of swarming motility and expansion of colonies on solid surfaces (Fig. 6). In order to verify that the increased expansion was a result of rhamnolipids the assay was repeated in the presence of the surfactant Tween 20. This resulted in a more than twofold increase in colony size of the reference strain. The presence of Tween 20 did not increase swarming motility of the rhamnolipid producing strain, most likely because of an abundant quantity of rhamnolipids compared to Tween 20.

### Continuous production of rhamnolipids in biofilm encased *P. putida*

**Fig. 7:**
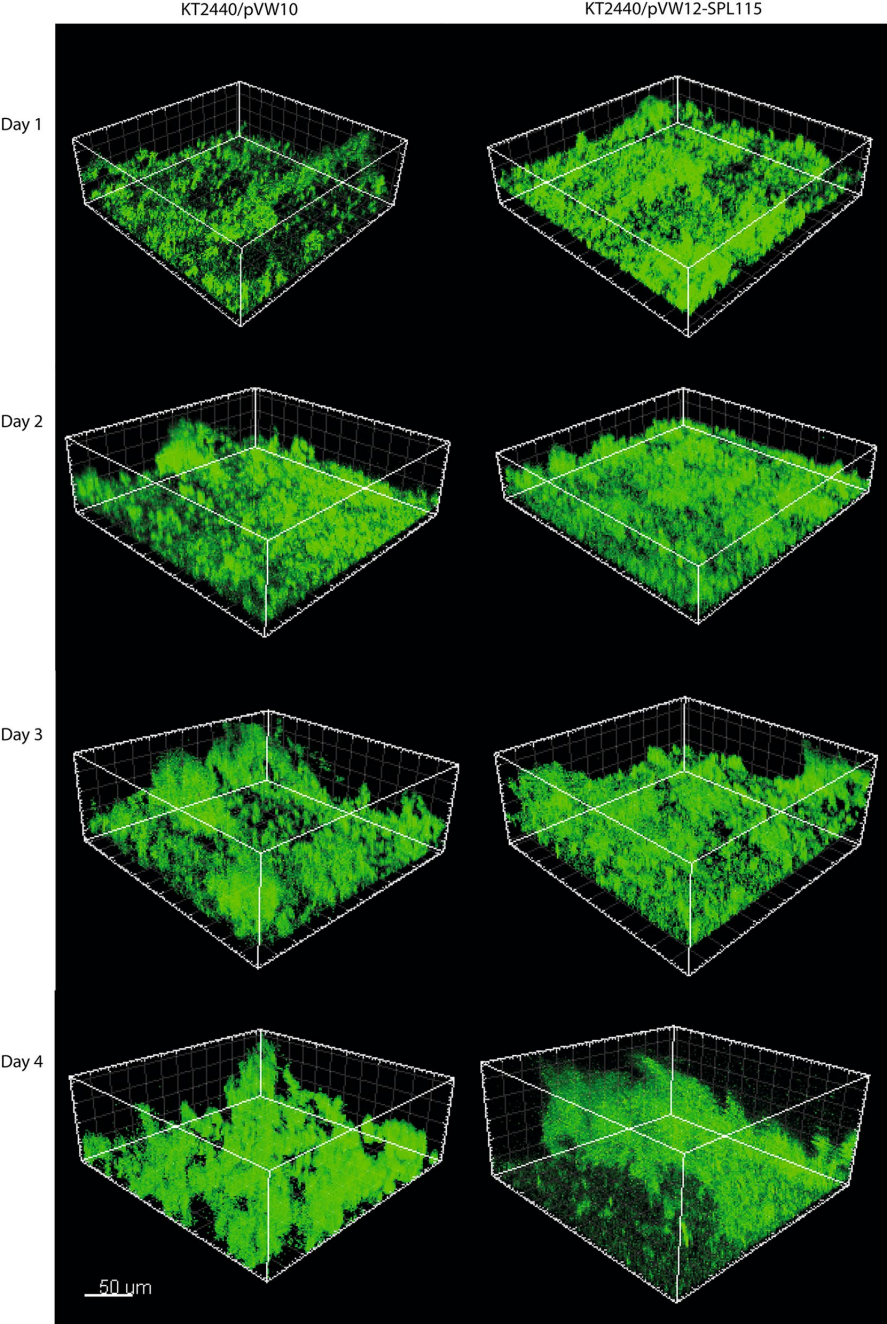
Biofilm development of a high rhamnolipid producing *P. putida* (KT2440/pVW12-SPL115) and the reference strain (KT2440/pVW10). Depicted are representative pictures of biofilm from the specified days. The *scale bar* indicates the size of 50 µm

**Fig. 8:**
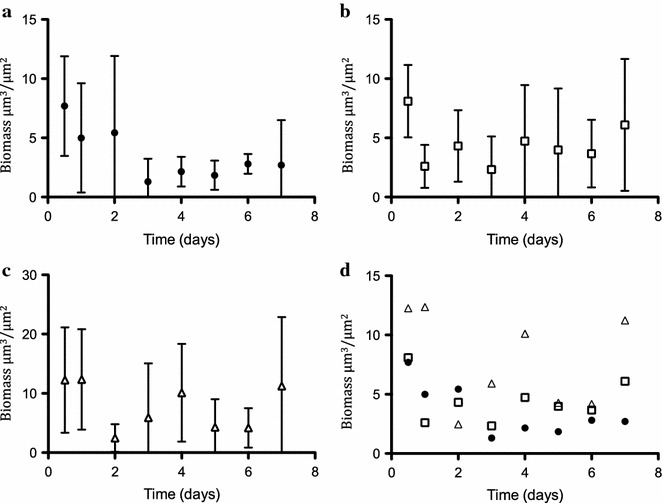
Biofilm biomass quantification. The biomass was quantified based on the obtained CSLM pictures. **a** The reference strain (KT2440/pVW10), **b** the medium rhamnolipid producer (KT2440/pVW12-SPL4), and **c** the high rhamnolipid producer (KT2440/pVW12-SPL115). In **d** strains are combined and only the mean values are depicted for clarity. The *symbols* represent the mean biomass and the *error bars* the standard deviation based on two biological replicates each composed of 7–11 biofilm pictures

**Fig. 9:**
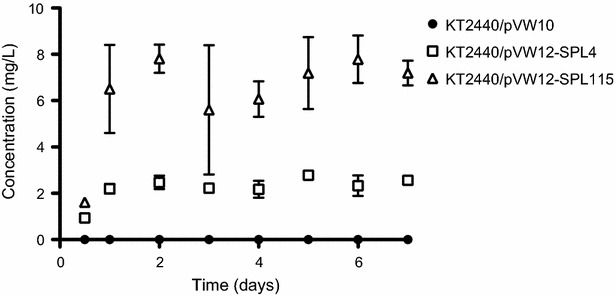
Continuous rhamnolipid production in a biofilm system. The reference strain (KT2440/pVW10), a medium (KT2440/pVW12-SPL4) and a high rhamnolipid (KT2440/pVW12-SPL115) producer were cultivated for 7 days and the rhamnolipid production quantified. The *symbols* represent mean concentration and the *error bars* the standard deviation of two biological replicates

A continuous production of rhamnolipids using biofilm as the production platform was evaluated using flow cell technology [28, 30]. The biofilm was grown on a glass substratum and growth was followed in situ using confocal laser scanning microscopy. A high and a medium rhamnolipid producer were grown and the rhamnolipid content quantified and compared to the reference strain. Representative pictures of the high rhamnolipid producer (KT2440/pVW12-SPL115) and the reference strain (KT2440/pVW10) are shown in Fig. 7. No apparent visual difference in the biofilm morphology was observed. However, the KT2440/pVW12-SPL115 biofilm appeared to contain more biomass. For quantitative comparison the biomass was quantified based on the obtained pictures using COMSTAT2 [31, 32]. Figure 8a, b and c show the quantified biomass of the biofilm from flow chambers. The fluctuations observed in the quantification demonstrate the heterogeneity and complexity of biofilm. The biofilm biomass is difficult to quantify, specially for *P. putida* biofilm [31]. Even though fluctuation occur a quantitative measure of the biomass enables comparison across the strains, particularly when combined with crystal violet assays. The biofilm stimulating effect observed in the microtiter assay (Fig. 5) is also evident in flow chambers (Fig. 8d). Although the biomass fluctuates the rhamnolipid production showed to be consistent and reaches a stabile level after which the production is constant for both the medium and the high rhamnolipid producer (Fig. 9). The rapid achievement of maximal production titter reflects a fast and reproducible establishment of a biofilm capable of producing rhamnolipids. Both the biofilm and the rhamnolipid production were stably maintained during the cultivation period.

## Discussion

The quest for replacing petrochemicals with biochemicals gives challenges in finding the optimal organism and cultivation conditions. In this study we have explored the potential of biofilms as an alternative production platform for biosynthesis of chemicals which are inherently troublesome to produce in conventional bioreactor setups.

### Heterologous biosynthesis and detection of rhamnolipids in *P. putida*

Although the fast and reliable method of using synthetic promoter libraries has been employed for several species [23, 24, 33, 34], this is the first time this technique has been employed for *Pseudomonas* ssp. The use of SPL made it possible to investigate the impact of heterologous rhamnolipid production in *P. putida* regarding growth rate and biofilm capabilities. We selected a constitutive rather than an inducible promoter design, as the even distribution of inducers into the biofilm could be hindered by biofilm specific factors such as the extracellular matrix. Importantly, we showed that rhamnolipids promotes biofilm formation, and constitutive production will not limit biofilm establishment (Figs. 5, 8).

Rhamnolipid production has been investigated in both *P. putida* and *P. aeruginosa* [17, 18]. Many of these studies have been made by indirect quantification and by measuring the total pool of rhamnolipids, although it is well known that different rhamnolipid congeners are made resulting in different physiochemical properties [4]. We aimed to investigate the applicability of biofilm as a production platform for rhamnolipids, for which reason it was crucial to develop a method by which the different congeners could be determined in case alterations in composition occurred as a consequence of perturbations in the intracellular metabolites. Furthermore, as production of rhamnolipids in a biofilm will result in low titters, a sensitive method for detection of small changes in rhamnolipid concentration was necessary.

With our method, we observed the fatty acid composition of the rhamnolipids to vary between C_8_ and C_12_ with C_10_ being the most abundant [4, 26]. This is similar to the rhamnolipids produced by *P. aeruginosa* [4, 26], and is expected due to the substrate specificity of *rhlA* from *P. aeruginosa* to C_10_ fatty acids [8]. The lower limit of detection of our method was 0.09 mg/L (data not shown) which is comparable to the method recently published by Rudden et al. [26].

### Biofilm as a production platform

Excessive foaming during rhamnolipid production in conventional bioreactors system remains a challenging problem in relation to maintaining sterility [19–21]. In addition to being a problem in maintaining sterility, foam formation can also result in disturbances of the culture broth and thereby result in decreased rhamnolipid production [20]. In this study we eliminated foam production by employing biofilm encapsulated cells in a flow system as production platform.

The biofilm mode of growth represent another set of challenges not present in other bioreactor systems. For example, since rhamnolipids are biosurfactants they could have a severe impact on the biofilm formation process by dissolving the encapsulated cells. Rhamnolipids could potentially be involved in removing extracellular polymeric substances thereby destabilising the biofilm and consequently disrupting it [27]. However, we found that rhamnolipids stimulated *P. putida* biofilm formation through enhanced cell motility as previously shown for *P. aeruginosa* [29]. However, the difference in biofilm morphology was not as pronounced in our *P. putida* biofilms as observed in *P. aeruginosa* [29]. We also observed that the amount of rhamnolipids has an effect on biofilm dispersal. The more rhamnolipids are being produced the earlier the biofilm dispersal occur. The presence of rhamnolipids does however result in more biofilm biomass compared to the reference strain in all cases (Fig. 5). Hence, there is an optimum for biofilm biomass and rhamnolipid content after which the biomass starts to decline.

Instability of biofilm cells has been reported in other studies as cell morphology did change during the cultivation time [35]. We verified the expression of the *rhlAB* genes in the biofilm by employing the plasmid encoded *gfp* reporter for visualisation of the biofilm in situ. In our case no changes were observed in relation to plasmid loss (data not shown), change in cell morphology or decreased rhamnolipid production.

In the present study we have focused on using biofilm as a platform for producing rhamnolipids and the associated challenges. Hence, no optimisations of the cells were made for increasing the production as well as for increased biofilm formation. This is a necessity in order to encounter the higher amount of rhamnolipids being produced in planktonic cultivation. A candidate for increasing production could be to reduce diversion of precursors from the biosynthesis of rhamnolipids. Wittgens et al. [18] eliminated the synthesis of polyhydroxyalkanoate as this competes for the rhamnolipid precursor HAA. Since this modification resulted in an increased yield, a similar approach could be the first step in order to achieve higher production titters in biofilm platforms. Wittgens et al. [18] also showed rhamnolipid synthesis to be growth independent and growth should be minimised for increasing the yield. We took advantage of this knowledge in using biofilm as the production platform for heterologous synthesis of rhamnolipids. The lowered growth activity of biofilm encased cells [22] should reflect an increased yield without hampering the biosynthesis. Another possibility is to increase productivity by increasing the precursors for rhamnolipid synthesis. This can be mediated by perturbing the intracellular energy levels and increase the ATP demand. Introducing the F_1_ part of the membrane bound F_0_F_1_ H^+^-ATP synthase increased the glycolytic flux in *E. coli* [36]. This may stimulate the intracellular processes and thereby increase the synthesis of rhamnolipids by stimulating the synthesis of precursors.

Other areas for further improvements of our production platform include prevention of biofilm dispersal by genetic engineering as previously described [28], and to integrate the production genes into the chromosome of the bacteria to eliminate possible loss of plasmids during production (although the employed plasmid can be stably maintained in *P. fluorescens* without selection [37]).

## Conclusion

In this study we successfully utilised biofilm as a production platform. By employing SPL we characterised the effect of producing rhamnolipids in relation to growth and on biofilm capabilities of the non-pathogenic bacterium *P. putida*. The in situ investigation of the biofilm formation enabled invaluable exploration of the effect of producing rhamnolipids. This study adds on to the increasing knowledge of *P. putida* and the wide applicability of this organism in industrial settings. The continuous production of rhamnolipids in a biofilm exemplified in this study show that this mode of growth could support production of troublesome biochemicals. A continuous production of biochemicals eliminate product inhibition and the inherent resistance of biofilm make it a competent candidate for producing biochemicals which are toxic as well as for using alternative feedstocks without being necessitated to make a detoxification.

## Methods

### Strains and growth conditions

*Escherichia coli* DH5α strain was used for standard DNA manipulations. *Pseudomonas aeruginosa* PAO1 and *P. putida* KT2440 were used for synthetic promoter library construction. The employed strains are listed in Table 1. The strains were propagated in modified lysogeny broth (LB) medium containing 4 g of NaCl/L instead of 10 g NaCl/L and with peptone instead of tryptone [38]. Biofilm experiments in flow chambers were made in modified FAB medium [31] supplemented with 1 mM sodium citrate. Biofilm inoculum was supplemented with 10 mM sodium citrate. Tetracycline concentration of 8 and 20 µg/mL was used for *E. coli* and *P. putida*, respectively. *Escherichia coli* and *Pseudomonas aeruginosa* were incubated at 37 °C and *P. putida* at 30 °C. The growth rates were determined in the exponential growth phase.

**Table 1:** Strains, plasmids and primers used in this study

Strain, plasmid or primer	Relevant characteristic or sequence	References
Strains		
*E. coli* DH5α	Host for plasmid maintenance	Laboratory strain
*P. putida* KT2440	Wild type strain; used for heterologous expression of *rhlAB* operon	[44]
*P. aeruginosa* PAO1	Wild type strain; used for amplification of *rhlAB* operon	[45]
Plasmids		
pBK-miniTn7-*gfp*2	Gm^r^; P_*rnB* P1_ *gfp*AGA Delivery vector for miniTn7-*gfp*2	[40]
pRK600	Cm^r^; *ori*ColE1 RK2-*mob* ^+^ RK2-*tra* ^+^ Helper plasmid in mating	[46]
pUX-BF13	Ap^r^; *mob* ^+^ *ori*R6K Helper plasmid providing Tn7 transposition function in trans	[47]
pJBA27	*gfp*mut3*	[48]
pME6031	Tc^r^; derivate of pME6010; cloning vector maintained in Pseudomonas strains without selection pressure	[37]
pVW10	pME6031::*gfp*mut3*	This study
pVW13	pVW10 with promoterless *rhlAB*	This study
pVW14	pVW10 with *rhlAB* and its native promoter	This study
pVW12-SPL1 to pVW12-SPL120	SPL in front of *rhlAB*	This study
Primers		
Gfp_Fwd	5′-AGATAGAATTCAGATTCAATTGTGAGCGG-3′	
Gfp_rev	5′-AGATACTGCAGTATCAACAGGAGTCCAAGC-3′	
SPL_rhlAB	5′-AGATAAGATCTACN_10_TTGACAN_16_TATAATN_6_CCCG-ATCGGCTACGCGTGAACA-3′	
rhlAB_rev	5′-AGATAGAATTCATCACAGCAGAATTGGCCC-3′	
SPL_con_rhlAB	5′-AGATAAGATCTATCGGCTACGCGTGAACA-3′	
Native_rhlAB	5′-AGATAAGATCTACCCTGCCAAAAGCCTGA-3′	

The restriction sites on the primers are indicated by underscore. N indicate a randomised base representing 25% of A, C, G or T

### Construction of synthetic promoter library

The reporter plasmid pVW10 was constructed by PCR amplification of *gfp*mut3* using Phusion polymerase and the primers Gfp_fwd and Gfp_rev, followed by double digestion of the fragment and vector pME6031 with *Eco*RI and *Pst*I. These were ligated together by T4 DNA ligase. The employed primers are listed in Table 1.

Constitutive SPL of the *rhlAB* operon was constructed as described by [23]. The SPL was based on putative rRNA promoters extracted from the genome sequence of *P. putida* KT2440 (accession number: NC_002947) and *P. aeruginosa* PAO1 (accession number: NC_002516). Promoters of varying strength were obtained by randomisation of the nucleotides surrounding −10 and −35 consensus sequences (primers: SPL_rhlAB and rhlAB_rev). The native promoter of the *rhlAB* operon in *P. aeruginosa* PAO1 and a promoterless *rhlAB* operon were made as reference to the constructed SPL and for validation of promoter activity (primers: Native_rhlAB, SPL_con_rhlAB and rhlAB_rev). The promoter sequence of the employed strains for investigating the correlation between *gfp* expression and growth rate, and for quantifying rhamnolipids has been listed in Additional file 1: Table S1. An alignment of the promoter sequences is provided in Additional file 1: Figure S3.

Genomic DNA from *P. aeruginosa* PAO1 was extracted using Wizard Genomic DNA Purification Kit (Promega). The *rhlAB* operon was PCR amplified from genomic *P. aeruginosa* PAO1 using Phusion polymerase. The employed primers are listed in Table 1. Following double digestion of the fragments containing *rhlAB* operon and vector pVW10 with *Bgl*II and *Eco*RI these were ligated together with T4 DNA ligase. All enzymes for DNA manipulations were bought from ThermoFisher Scientific and used as recommended. Primers were purchased from Integrated DNA Technologies. The SPL_rhlAB primer was purchased as ultramer.

The SPL was introduced into *P. putida* by electroporation [39] to avoid biases in promoter strength by using *E. coli* as an intermediate host. The SPL was constructed in three independent rounds. *Pseudomonas putida* KT2440 containing SPL were randomly picked followed by manual inspection for highly active promoters missed in the random selection.

For biofilm formation in flow chambers the reference strain containing *P. putida* pVW10 was fluorescently tagged at an intergenic neutral chromosomal locus with *gfp* in mini-Tn7 construct as previously described [40]. The rhamnolipid producing mutants were visualised by the plasmid encoded *gfp* reporter gene.

### GFP analysis

The promoter strength was determined by quantification of *gfp* expression at single-cell level by flow cytometry. The *P. putida* strains containing the promoter library were grown in LB medium supplemented with tetracycline in 96-well microtiter plates. Overnight cultures of approximately 16 h were diluted 50-fold and incubated for additionally 3 h at 30 °C with shaking at 600 rpm to ensure exponentially growth. The cultures were diluted five times in 0.9% NaCl before measuring *gfp* expression on a FACSCalibur (Becton–Dickinson) flow cytometer. The data was analysed in FlowJo. For correlation of *gfp* expression and rhamnolipid quantification the cells were grown in shake flasks.

### Rhamnolipid extraction and analysis

A mono-rhamnolipid standard was used for calibration and validation purposes (Sigma-Aldrich). Samples were drawn from the culture broth for rhamnolipid quantification in planktonic cells. Rhamnolipid quantification from biofilm encased cells was made from the effluent of the flow chambers. Cells were removed by filtration (0.22 µM) and the supernatant, 1 mL, was mixed with two times the volume of 70% acetonitrile. A two-phase separation was achieved by addition of a mixture of sodium sulphate and sodium chloride powder ≈100 mg followed by centrifugation at 4500×*g* for 5 min (QuEChER extraction) [41]. Chloramphenicol (50 mg/mL in ethanol) was added to a final concentration of 10 µg/mL as internal standard. The organic phase was transferred to HPLC vials and analysed on a reverse phase UHPLC-HRMS on a maXis HD quadrupole time of flight (qTOF) mass spectrometer (Bruker Daltonics, Bremen, Germany) connected to an Ultimate 3000 UHPLC system (Dionex, Sunnyvale, CA, USA) equipped with a 10 cm Kinetex C_18_ column (Phenomenex Torrance, CA, USA) running a linear 10–100% gradient for 15 min at 40 °C and a flow at 0.4 mL/min [42]. The qTOF was operated in ESI^−^ negative mode, scanning *m*/*z* 50–1300, with alternating fragmentation energy over the quadrupole of 0 and 25 eV, each for 0.25 s.

### UHPLC-HRMS data analysis

Extracted ion chromatograms (±2 mDa) of the [M–H]^−^ ions were constructed from the 0 eV volt data using Compass TargetAnalysis (version 1.3 Bruker Daltonics), which also verified the isotopic patterns (I-fit < 50). The 25 eV volt data were used to verify identity of the rhamnolipids, as fragment ions of rhamnose and one fatty acid moiety cold be identified by the loss of the fatty acid distant to the rhamnose molecule (Additional file 1: Figure S4). The retention time (±0.02 min) was compared to an authentic standard (Sigma-Aldrich). The rhamnolipid standard contained according to the manufacture 90% mono-rhamnolipid and 5% di-rhamnolipid, each of these contained several congeners with different fatty acid chains. Each of the mono-rhamnolipid congeners were integrated, [M–H]^−^, and their fractions determined in the purchased standard. The standard contained the following fractions of congeners: 84.1% Rha-C_10_-C_10_, 9.7% Rha-C_8_-C_10_ to; 3.4% Rha-C_10_-C_12:1_, 2.5% Rha-C_10_-C_10:1_ and 0.3% Rha-C_10_-C_12_.

Calibration of rhamnolipids was made in acetonitrile–water (1:1 v/v) dilutions. The peak areas of rhamnolipids were normalised to that of chloramphenicol [M–H]^−^ (10 µg/mL). The standards were made in steps of 0.2 µg/mL from 0.5 to 1.7 µg/mL and from 1.7 to 30 µg/mL in steps of 2 µg/mL. Limit of detection was determined as the lowest point where a peak with s/n of 15 could be detected. Limit of detection was 0.09 µg/mL. R^2^ of 0.995 was obtained in the range 0.09–12 µg/mL of the standard.

### Swarming motility

Swarming motility was assayed in LB medium containing 20 µg/mL tetracycline and solidified with 0.5% agar. The plates were point inoculated at the surface and incubated for 24 h at 22 °C.

### Crystal violet biofilm assay

Quantification of biofilm formation in static microtiter dishes was made by crystal violet staining as described by O’Toole and Kolter [43]. Briefly, over night cultures were diluted to an OD_600_ of 0.010 and inoculated in 100 µL LB supplemented with 20 µg/mL tetracycline for the specified time. The wells were emptied and washed with 0.9% NaCl followed by 15 min of staining with 0.1% crystal violet (Sigma-Aldrich). The wells were washed twice in saline and adhered crystal violet was subsequently solubilised in 96% ethanol for 15 min before quantification by spectrometry at Abs_595_. Microtiter dished were made of polystyrene (TPP Techno Plastic Products AG).

### Biofilm cultivation in flow chambers

Biofilms were cultivated in three-channel flow chambers with individual channel dimensions of 1 × 4 × 40 mm covered with glass coverslip (Knittel 24 × 50 mm) serving as substratum for biofilm formation. The system was prepared and assembled as previously described by Tolker-Nielsen and Sternberg [30]. Overnight cultures were made in modified FAB medium supplemented with 10 mM sodium citrate. The overnight cultures were diluted to an OD_600_ of 0.010 and aliquots of 500 µL were inoculated into each channel of the flow chambers. Bacterial attachment was allowed for 1 h with the chambers turned upside down without flow. The flow systems was incubated at 22 °C with a laminar flow rate of 3 mL/h obtained by a Watson Marlow 205S peristaltic pump. Modified FAB medium supplemented with 1 mM of sodium citrate was used for biofilm cultivation [31]. Tetracycline was added to the growth medium for plasmid maintenance. Bacterial growth upstream of the flow channels were removed by cutting the affected part of the tubing under sterile conditions and reattached the tubes to the flow channel.

### Microscopy and image processing

Biofilm was followed in situ using a Leica TCS SP5 confocal laser scanning microscope equipped with detectors and filters set for monitoring Gfp fluorescence. Images were obtained with a 63×/1.20 water objective. Images of the biofilm were taken at the specified time points. Images were all acquired from random positions at a distance of 5–10 mm from the inlet of the flow channels. Biomass of the biofilm was determined based on the acquired pictures by employing COMSTAT2 [31, 32]. Simulated three-dimension images were generated using the Imaris software package (Bitplane AG).

## Additional file

**Additional file 1.** Supporting information with additional information on SPL and describing the structural identification of the rhamnolipid congeners based on fragmentation patterns.

## Abbreviations

Gfp: green fluorescent protein
HAA: 3-(3-hydroxyalkanoyloxy)alkanoate
HRMS: high resolution mass spectrometry
LB: lysogeny broth
MS/HRMS: tandem mass spectrometry
Rha-Rha-C_10_-C_10_: l-Rhamnosyl-l-rhamnosyl-3-hydroxydecanoyl-3-hydroxydecanoate
SPL: synthetic promoter library
UHPLC: ultra performance liquid chromatography
