# Supplementary material for: Biofilm as a production platform for heterologous production of rhamnolipids by the non-pathogenic strain Pseudomonas putida KT2440
Source: Microb Cell Fact. 2016 Oct 24;15:181. doi: 10.1186/s12934-016-0581-9 (PMC5075983; doi:10.1186/s12934-016-0581-9)
Supplement: Supplementary file 1 — Additional file 1. Supporting information with additional information on SPL and describing the structural identification of the rhamnolipid congeners based on fragmentation patterns. [file 12934_2016_581_MOESM1_ESM.docx]

**Supporting information for**

**Biofilm as a production platform for heterologous production of rhamnolipids by the non-pathogenic strain *Pseudomonas putida* KT2440**

Vinoth Wigneswaran^1^ ([vw@bio.dtu.dk](mailto:vw@bio.dtu.dk)), Kristian Fog Nielsen^1^ ([kfn@bio.dtu.dk](mailto:kfn@bio.dtu.dk)), Claus Sternberg^1^ ([cst@bio.dtu.dk](mailto:cst@bio.dtu.dk)), Peter Ruhdal Jensen^2^ ([perj@food.dtu.dk](mailto:perj@food.dtu.dk)), Anders Folkesson^3^ ([afol@vet.dtu.dk](mailto:afol@vet.dtu.dk)), Lars Jelsbak^1*^ ([lj@bio.dtu.dk](mailto:lj@bio.dtu.dk))

^1^ Department of Systems Biology, Technical University of Denmark, 2800 Kgs. Lyngby, Denmark

^2^ National Food Institute, Technical University of Denmark, 2800 Kgs. Lyngby, Denmark

^3^ National Veterinary Institute, Technical University of Denmark, 1870 Frederiksberg C, Denmark

*Corresponding author: DTU Systems Biology, Building 301, DK2800, Denmark, [lj@bio.dtu.dk](mailto:lj@bio.dtu.dk), +4545256129

**Synthetic promoter library**

The promoter strengths in the synthetic promoter library determined by the Gfp intensities are shown in an enlarged version in Fig. S1.

**Metabolic load**

Initial screening were made in order to investigate the metabolic load of producing rhamnolipids by determining the growth rate of a selection of strains with various rhamnolipid producing capabilities based on *gfp* expression levels (Fig. S2). The rhamnolipid production leads to a decrease in growth rate. Hence, the production imposes a metabolic load on the cells. The outlier in Fig. S2 is likely owing to a deletion in the *rhlAB* operon as a consequence of the metabolic load which leads to a growth rate similar to the strains not producing rhamnolipids.

**Promoter sequences**

The promoter sequences of the employed promoters are listed in Table S1. The promoter sequences have been aligned to the randomised promoter sequence used to make the SPL (Fig. S3). As expected the randomisation have resulted in a diverse set of sequences which give rise to the variability of the promoter strengths.

**Rhamnolipid identification**

Identification of the rhamnolipid congeners was based on their elementary composition. For identification of any possible rhamnolipid congeners in a sample, a list containing different combinations of rhamnose and fatty acids with varying chain length and saturation was used for screening the chromatograms (Table S2). However, the engineered *P. putida* host only produced a few different congeners (Fig. S4a). The chromatograms are based on the obtained mass spectrum (Fig. S4b) which shows the determined *m/z* values.

The abovementioned screening method suffers from the lack of ability to discriminate between isomers, e.g. between Rha-C_12_-C_10_ and Rha-C_10_-C_12_. For this reason pseudo MS/HRMS (MS-E) was made for structural identification of the different congeners. The fragmentation pattern of the rhamnolipid congeners revealed the fatty acid composition and was used for the identification (Table S2 and Fig. S4c,d). An example of fragmentation is shown for Rha-C_10_-C_10_ in Fig. S4d. The fragmentation point is indicated resulting in a *m/z* 333.1913 ion which is apparent in the 25 eV mass spectrum together with the lost fatty acid moiety of *m/z* 169.1236. In this way both the length of the fatty acid and their mutual position was elucidated. The obtained results correspond with previous results [[1](#_ENREF_1), [2](#_ENREF_2)].

**Reference List**

1. Rudden M, Tsauosi K, Marchant R, Banat IM, Smyth TJ: **Development and validation of an ultra-performance liquid chromatography tandem mass spectrometry (UPLC-MS/MS) method for the quantitative determination of rhamnolipid congeners.** *Appl Microbiol Biotechnol* 2015, **99:**9177-9187.

2. Deziel E, Lepine F, Milot S, Villemur R: **Mass spectrometry monitoring of rhamnolipids from a growing culture of Pseudomonas aeruginosa strain 57RP.** *Biochim Biophys Acta* 2000, **1485:**145-152.

**Figures**

**Fig. S1** Rank ordered promoter strengths in the synthetic promoter library determined by the Gfp intensities. The names of the constructed plasmids are shown on the X-axis.

**Fig. S2** Investigation of the correlation between *gfp* expression and growth rate. The growth rate decrease as the *gfp* expression increases.


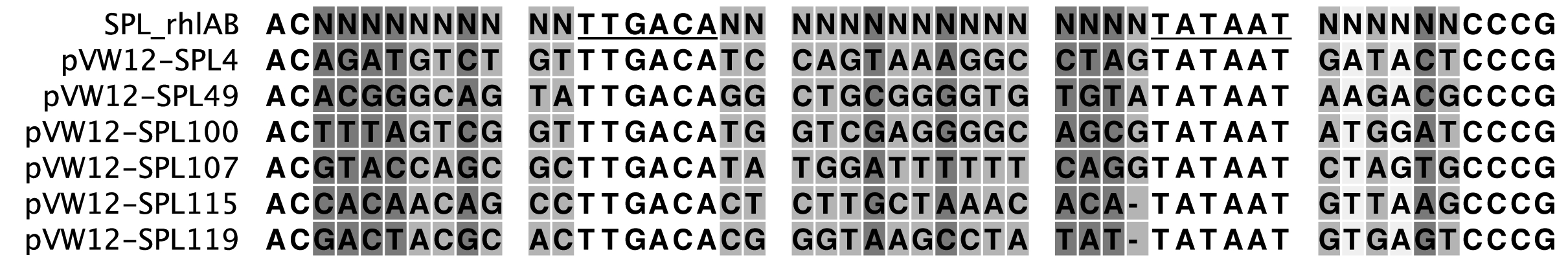


**Fig. S3** Alignemnt of the promoter sequences used in this study. The first line indicate the randomised promoter sequence followed by the promoters used in this study. The varying nucleotides are highlighted in grey boxes. The lighter the boxes the more conserved are the bases. The -10 and -35 regions are underlined.

**Fig. S4** UHPLC-HRMS analysis of rhamnolipids. Picture **a** is the extracted ion chromatogram of the internal standard and the four most abundant rhamnolipid congeners from the engineered *P. putida* strain KT2440/ pVW12-SPL115. The first peak is chloramphenicol followed by the rhamnolipid congeners Rha-C_8_-C_10_, Rha-C_10_-C_10_, Rha-C_10_-C_12:1_ and Rha-C_12_-C_12_. The full scan mass spectrum of the most predominant rhamnolipid congener Rha-C_10_-C_10_ is shown in figure **b**. The 25 eV mass spectrum of Rha-C_10_-C_10_ is depicted in figure **c**. In figure **c** the [M-H]^-^ ion and the fragmented ion can be seen from the pseudo MS/MS analysis. An example of the fragmentation pattern is shown in figure **d** for Rha-C_10_-C_10_. The breaking point and the resulting fragment masses are indicated in red.

**Tables**

**Table S1** Promoter sequence of the employed strains. The – indicate the deletion of a nucleotide compared to the randomised promoter.

| Strain | Sequence |
| --- | --- |
| KT2440/pVW12-SPL4 | ACAGATGTCTGTTTGACATCCAGTAAAGGCCTAGTATAAT  GATACTCCCG |
| KT2440/pVW12-SPL49 | ACACGGGCAGTATTGACAGGCTGCGGGGTGTGTATATAAT  AAGACGCCCG |
| KT2440/pVW12-SPL100 | ACTTTAGTCGGTTTGACATGGTCGAGGGGCAGCGTATAAT  ATGGATCCCG |
| KT2440/pVW12-SPL107 | ACGTACCAGCGCTTGACATATGGATTTTTTCAGGTATAAT  CTAGTGCCCG |
| KT2440/pVW12-SPL115 | ACCACAACAGCCTTGACACTCTTGCTAAACACA-TATAAT  GTTAAGCCCG |
| KT2440/pVW12-SPL119 | ACGACTACGCACTTGACACGGGTAAGCCTATAT-TATAAT  GTGAGTCCCG |

**Table S2** The rhamnolipid congeners screened in the chromatograms. Pseudomolecular ions and fragmentation ions used for structural identification of the different rhamnolipid congeners is shown. The MS/MS ions are the fragmented ions. The first ion is the rhamnose and one fatty acid residue and the next is the fatty acid ion. The ions marked with * could be identified in the MS/MS (25eV mass spectrum). The remaining ions could not be identified due to the absence of the congener or very low presence.

| **Compound** | **MS**  **[M-H]^-^** | **MS/MS**  **[M-H]^-^** |
| --- | --- | --- |
| Rha-C_10_-C_10_ | 503.3226 | 333.1925* - 169.1239* |
| Rha-C_10_-C_12_ | 531.3539 | 333.1925* - 197.1549* |
| Rha-C_10_-C_12:1_ | 529.3382 | 333.1925* - 195.1390* |
| Rha-C_8_-C_10_ | 475.2913 | 305.1603* - 169.1239* |
| Rha-C_12_-C_12:1_ | 557.3695 | 360.2148 – 197.1542 |
| Rha-C_12_-C_12_ | 559.3852 | 360.2148 – 199.1698 |
| Rha-C_10_-C_10:1_ | 501.3069 | 333.1925 – 167.1083 |
| Rha-C_8_-C_8_ | 447.2594 | 305.1603 – 143.1072 |
| Rha-C_8_-C_8:1_ | 445.2437 | 305.1603 – 141.0916 |
| Rha-C_8_-C_10:1_ | 473.2750 | 305.1603 – 169.1229 |
